# Ultrafast laser printing of self-organized bimetallic nanotextures for multi-wavelength biosensing

**DOI:** 10.1038/s41598-018-34784-y

**Published:** 2018-11-07

**Authors:** D. Pavlov, S. Syubaev, A. Cherepakhin, A. Sergeev, O. Vitrik, A. Zakharenko, P. Danilov, I. Saraeva, S. Kudryashov, A. Porfirev, A. Kuchmizhak

**Affiliations:** 1 School of Natural Sciences, Far Eastern Federal University, 690041 Vladivostok, Russia; 2 Institute of Automation and Control Processes, Far Eastern Branch, Russian Academy of Science, Vladivostok, 690041 Russia; 3 ITMO University, St. Petersburg, 197101 Russia; 4 Lebedev Physical Institute, Russian Academy of Sciences, Moscow, 119991 Russia; 5 National Research Nuclear University MEPhI, Moscow, 115409 Russia; 6 Samara National Research University, 34 Moskovskoe Shosse, Samara, 443086 Russia; 7 Image Processing Systems Institute of the RAS-Branch of FSRC “Crystallography & Photonics” of the RAS, 151 Molodogvardeyskaya St., Samara, 443001 Russia

## Abstract

Surface-enhanced spectroscopy (SES) techniques, including surface-enhanced photoluminescence (SEPL), Raman scattering (SERS) and infrared absorption (SEIRA), represent powerful biosensing modalities, allowing non-invasive label-free identification of various molecules and quantum emitters in the vicinity of nanotextured surfaces. Enhancement of multi-wavelength (vis-IR) excitation of analyte molecules of interest atop a single textured substrate could pave the way toward ultimate chemosensing performance and further widespread implementation of the SES-based approaches in various crucial areas, such as point-ofcare diagnostics. In this paper, an easy-to-implement ultrafast direct laser printing via partial spallation of thermally-thick silver films and subsequent large-scale magnetron deposition of nanometer-thick Au layers of variable thickness was implemented to produce bimetallic textured surfaces with the cascaded nanotopography. The produced bimetallic textures demonstrate the strong broadband plasmonic response over the entire visible spectral range. Such plasmonic performance was confirmed by convenient spectroscopy-free Red-Green-Blue (RGB) color analysis of the dark-field (DF) scattering images supported by numerical calculations of the electromagnetic (EM) “near-fields”, as well as comprehensive DF spectroscopic characterization. Bimetallic laser-printed nanotextures, which can be easily printed at ultrafast (square millimeters per second) rate, using galvanometric scanning, exhibited strong enhancement of the SEPL (up to 75-fold) and SERS (up to 10^6^ times) yields for the organic dye molecules excited at various wavelengths. Additionally, comprehensive optical and sensing characterization of the laser-printed bimetallic surface structures allows substantiating the convenient spectroscopy-free RGB color analysis as a valuable tool for predictive assessment of the plasmonic properties of the various irregularly and quasi-periodically nanotextured surfaces.

## Introduction

Surface plasmons, resonant oscillations of the electron-ion plasma in the noble-metal nanostructures induced by irradiating photons, are known to provide substantial augmentation of the electromagnetic (EM) field localized near interfaces of the metals with their dielectric environment^1^. Development of surface structures and nanotextured surfaces supporting plasmon-mediated EM-field enhancement in a broad spectral range, covering whole visible spectrum, as well as its near infrared (near-IR) part, is of mandatory importance for various crucial applications^2–4^. In particular, such textured surfaces with superbroadband plasmonic response can be used to build novel high-performance optoelectronic devices^5,6^, solar-cell elements^7^, as well as to develop versatile and flexible biosensing platforms based on various surface-enhanced spectroscopy (SES) processes - surface-enhanced Raman scattering (SERS), photoluminescence (SEPL) and infrared absorption (SEIRA)^8–11^.

As routine SES-based biosensing experiments require expendable nanotextured substrates for various analyte types to provide accurate measuring information, there is a great demand for high-performance economically justified strategies for such substrate fabrication. In the past decade, direct processing of different noble-metal materials using short- and ultrashort laser pulses has become a mature technology, allowing rapid fabrication of various nanotextured morphologies. Currently, pulse repetition rate of commercially available laser systems used in such experiments enters sub-GHz range^12,13^ ensuring extremely fast material processing at the speed, reaching several cm^2^ per second. Several laser-based approaches were efficiently implemented to obtained various nanopatterned surfaces, exhibiting strong plasmonic response. Such approaches include the formation of the laser-induced periodic surface structures^14^, direct surface texturing via ablative phenomena^15–19^, deposition of the nanoparticles resulting from laser-induced spallation of bulk targets^20,21^, laser-induced forward transfer^22–24^, etc. However, even though good biosensing performance was reported for certain types of laser-fabricated textures, such surfaces exhibit either a low density of the plasmon-related hot spots per unit area or low spot-to-spot reproducibility of the measured signals. Moreover, none of them were certified or optimized so far to exhibit broadband plasmonic response over the whole visible spectral range. Noteworthy, the ability to harness multi-wavelength excitation within a single substrate, which can be produced in a controlled manner using some inexpensive fabrication technology, paves the way toward achieving ultimate biosensing performance and further widespread implementation of the SES-based approaches in various crucial areas as point-of-care diagnostics.

Here, we propose an easy-to-implement double-step approach for fabrication of bimetallic surfaces with cascaded nanotopography, exhibiting strong and broadband plasmonic response. The proposed approach is based on the direct ultrafast femtosecond (fs) laser spallation of the Ag-film surface followed by the deposition of the nanometer-thick layer of the other plasmonic metals, such as Au, Pt or Al. The direct fs-laser ablation of the Ag film by moderately focused pulses produces the surface craters uniformly covered by self-organized nanospikes, while their non-uniform coverage with the nanometer-thick layer of the other noble-metal material produces the cascaded bimetallic nanotopography. The resulting bimetallic textures demonstrate strong broadband plasmonic response over the whole visible spectral range, which is confirmed by supporting calculations of the EM “near fields” as well as comprehensive optical characterization by means of dark-field (DF) scattering microspectroscopy and accompanying RGB color analysis of the DF scattering images. The strong plasmonic response of the bimetallic laser-printed nanotextures is shown to provide considerable enhancement of the corresponding SEPL (up to 75-fold) and SERS (up to 10^6^) yields from Rhodamine 6G (R6G) organic dye molecules photo-excited at different wavelengths. The spectrally broadband and rather strong plasmonic response of the bimetallic spiky textures, which can be easily printed at high (square millimeter per second) rate using the galvanometric scanning techniques, make the proposed approach promising for various routing biosensing applications.

## Methods

### Fabrication of the superbroadband bimetallic self-organized nanotextures

The 500-nm thick Ag films were first deposited onto pre-cleaned silica glass substrates using a magnetron sputtering procedure (Kurt Lesker) at sputtering rate of 50 nm⋅min^−1^. The fabricated films were then textured by second-harmonic (*λ* = 515 nm) fs (≈300 fs) pulses generated by a fiber laser system (Satsuma, Amplitude System), supporting MHz-level pulse repetition rate. The laser pulses were focused in the air with microscope objectives of different numerical apertures NA = 0.13, 0.3, 0.65, 0.95, allowing to vary gradually the diameter of laser irradiated spot in the range from 18.4 to 0.35 *μ*m^2^, respectively. For the certain experiments, the linearly-polarized Gaussian-shaped output laser beam was converted into donut-shaped one, using a commercial radial polarization converter (S-waveplate, Altechna^25^). Laser fabrication procedure was performed by scanning the sample surface with the laser beam. To do this, either the translation of the sample with a precise 3D nanopositioning platform (Newport XM and GTS series) or the ultrafast beam scanning with a galvanometric scanner (ATEKO-TM, scan speed up to 7 m/s, 100-mm focal-length anti-reflective objective with a 100-mm wide view field and waist 1/e-diameter of 30 *μ*m) synchronized to the laser system, were performed^26,27^.

Finally, Au films of variable thickness ranging from 25 to 100 nm were deposited above the laser-textured areas, using a magnetron deposition at a sputtering rate of 25 nm⋅min^−1^. The sample holder was rotated at a constant speed of 25 rpm to ensure uniform deposition. The morphology of the laser-textured surface spots before and after the deposition of the capping Au films was characterized by high-resolution scanning electron microscopy (SEM, Carl Zeiss Ultra 55+). Additionally, several other materials, such as Pt and Al, were also coated as thin films above the Ag textures to check their deposition and coverage features (see Fig. S1 in Supporting information).

### Dark-field microspectroscopy and RGB color analysis

DF backscattered images of the self-organized laser-textured surfaces were acquired upon their side illumination (at 70° with respect to the sample normal) by s-polarized white-light radiation from a stabilized calibrated tungsten bulb (HL2000-HP, Ocean Optics). The DF backscattered images were collected by means of two long-focal-distance lenses with NA = 0.42 and 0.8 to provide variable collection angle of the backscattered radiation. The DF images were captured by a charge-coupled device (CCD) camera (DS-Ri1 Digital sight, Nikon). The corresponding back-scattering spectra were measured, using a home-built confocal system, comprising the abovementioned objectives and a grating-type spectrometer (Shamrock 303i, Andor) equipped with a sensitive thermoelectrically-cooled camera (TE-CCD, Newton 971) to analyze the spectrum. One adjustable pinhole was used to tune the acquisition area size.

To perform RGB color analysis, the recorded DF backscattered images were analyzed and decomposed into the corresponding color channels, using ImageJ software (NIH). An IR-cutting filter (Thorlabs GS800) was inserted into the optical scheme to block near-IR radiation in the DF images.

Additionally, the optical properties of the fabricated textured surfaces in the near-IR spectral region were probed using a Fourier-transform IR spectrometer (Vertex-80, Bruker) coupled to an infrared microscope (Hyperion 1000, Bruker). All measurements were performed in the reflection mode with a Cassegrain lens (NA = 0.5) by averaging 1500 scans at the spectral resolution of 8 cm^−1^ for the 50 randomly chosen areas patterned under the same laser-fabrication conditions. The signal acquisition area was adjusted using a knife-edge aperture placed in the image plane. The reflection from the bulk silver substrate was used for calibration. For the textured areas printed at various laser spot sizes, the actual size of the patterned vs unmodified areas was taken into account.

### FDTD calculations

Finite-difference time-domain calculations (FDTD, Lumerical Solutions package) were undertaken to assess approximate enhancement of the EM field amplitude near the isolated spikes. Taking into account the real nanotopography of the spallative crater, we considered a single silver nanospike with its height of 300 nm, diameter of 60 nm and a round tip of 30-nm curvature radius. High-resolution SEM images of the isolated spikes were used to further model the non-uniform coverage of the Ag spike with the Au capping layer of the equivalent thickness of 25, 50 and 100 nm. The modeled structures were excited with a linearly-polarized Gaussian source and wavelengths of 405, 532 and 632 nm. The size of the elementary Yee cell was fixed at 1 × 1 × 1 nm^3^, while the perfectly matched layers were used to limit the computation volume. The dielectric functions of Ag and Au materials were modeled with a built-in software, fitting the tabulated reference data from^28^.

### SEPL and SERS measurements

To demonstrate the multi-wavelength biosensing performance of the laser-printed textures, the nanometer-thick layer of R6G molecules was deposited onto the tested sample. To do this, the sample with the textured areas and the various thicknesses of the Au capping layer was immersed into 10^−6^ M R6G ethanol solution for 1 h, then rinsed in the distilled water and dried by pressurized gaseous nitrogen. SEPL signal from the R6G layer covering the textured and smooth areas of the sample was first analyzed with a confocal laser scanning microscope (LSM 800, Carl Zeiss) equipped with a 20x dry lens (Plan-Apochromat 20x/0.8 M27) and an Airyscan detection unit^29^. The microscope allows switching between four diode laser sources, emitting at the wavelengths centered at 405, 488, 561 and 630 nm. Measurements were performed in the Airyscan mode with a scanning area of 50 × 50 *μ*m^2^ providing the nominal pixel size of 70 nm. Depending on the wavelength of the used laser source, the excitation intensity was adjusted to maintain the reasonable signal-to-noise ratio and account for the spectrally dependent excitation efficiency of R6G molecules (see Fig. 4(e)). For all these wavelengths, the intensity level was adjusted to avoid photodegradation of the molecules even after several measurement cycles.

To measure the SEPL/SERS spectra from the R6G molecules, we utilized the same home-build confocal setup used in this study for the DF microspectroscopy. The R6G PL was pumped with a linearly polarized 532-nm CW laser source (Melles Griot) focused onto the sample surface by a 0.42-NA lens. The size of the laser spot in the focal plane was adjusted with a beam expander to fit the diameter of the isolated self-organized spallative texture. To compare reproducible SEPL yields, the PL spectra obtained on the mono- and bimetallic textured surfaces as well as on the smooth Ag film and glass slide were averaged over 50 similar spectra taken from the randomly chosen spallative textures. Specifically, for SERS measurements, the laser radiation was focused with a dry lens (NA = 0.8) and then similar averaging procedure was performed.

## Results and Discussions

### Ultrafast fabrication of bimetallic nanotextures

**Figure 1:**
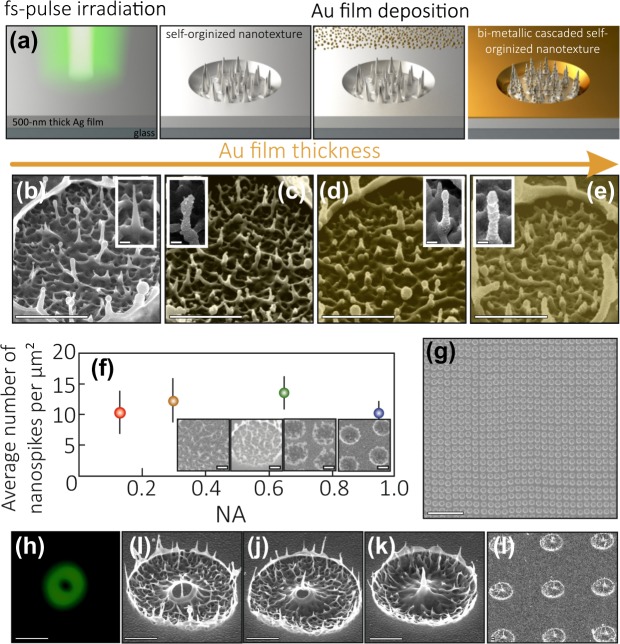
Summary of the fabrication procedure of the bimetallic Ag-Au cascaded nanotextures. (**a**) Direct laser ablation of the “thermally thick” Ag film surface is followed by the magnetron deposition of the nanometer-thick Au capping layer. (**b**–**e**) Series of the side-view SEM images illustrating the evolution of the nanotopography inside the single spallative crater upon increasing the thickness of the Au capping layer. The thickness of the deposited Au layer is 0 (**b**), 25 (**c**), 50 (**d**) and 100 nm (**e**). The spallative craters were produced at pulse fluence of 2 J/cm^2^. Close-up view of the single spiky structure (insets) shows their non-uniform coverage with the Au capping layer producing the cascaded nanoscale texture. Scale bars on the main images and insets correspond to 1 *μ*m and 50 nm, respectively. (**f**) Averaged density of the spiky structures in the craters N versus the NA of the focusing lens. (**g**) Large-scale array of the spallative craters printed using the fast laser-beam scanning with the galvanometric device. Scale bar corresponds to 20 *μ*m. (**h**) Focal-plane intensity distribution of the donut-shape beam. (**i**–**k**) Series of the side-view SEM images showing the surface evolution upon irradiation with a donut-shape beam. (**l**) SEM image showing the array of the spallative craters with the central micron-size protrusion printed with a donut-shape beam. Scale bars in images (**h**–**l**) correspond to 1.5 *μ*m.

Spiky nanostructures almost uniformly distributed inside the crater appear on Ag film surface upon single-shot fs-pulse irradiation at pulse energy slightly above the spallation threshold ≈1.4 ± 0.2 J/cm^2^ (Fig. 1(a,b)). The boundary rim of the spallative crater typically contains larger nanotips. The formation of such texture occurs via melting of the upper film layer and its subsequent ejection through the relaxation of the laser-generated stress^16,30–32^. The thickness of the molten film layer repeating to some extent in the lateral direction the incident Gaussian-like intensity distribution increases in depth versus the applied pulse energy. When the laser-driven molten front reaches the substrate-film boundary, one can observe the undesirable “hybrid” ablation behavior. In particular, the central part of the spallative crater detaches from the underlying substrate as a whole, forming the microbump^16^. In this respect, the irradiated metal film should be thick enough to maintain the spallation or “bulk-like” behavior^30^ within the fixed range of the above-threshold fluencies (below we will use the term “thermally thick” film).

More pronounced nanotopography can be obtained using magnetron deposition of the thin metal film above the laser-printed spallative structures as illustrated by the series of the highly magnified SEM images (Fig. 1(c–e)). As one can see, owing to a large surface area of the laser-printed spiky craters, the deposited Au film non-uniformly covers their surface producing the cascaded bimetallic nanotopography (the randomly arranged Ag tips covered with the Au nano-flakes).

The nanoscale topography of the produced bimetallic surface features generally depends on the thickness of the capping Au layer. Alternatively, the variation of the magnetron deposition rate is found to weakly affect the resulting nanoroughness. In this way, it is important to carefully optimize the first fabrication step (namely, laser spallation) in terms of the maximal density of the spiky structures maintaining, at the same time, their uniform distribution. As the spallation process proceeds on the surface of the thermally thick film, its thickness does not affect the resulting nanotopography. In this way, we performed the systematic studies of the effect of the surface nanotopography on the focusing conditions of the laser radiation controlled by the NA of the objective lens (see Methods for details). For all focusing conditions, we considered the fluence slightly above the spallation threshold which was found to provide rather uniform nanotextured surfaces of the spallative craters optimal in terms of biosensing applications^16,33^. The average density of spiky features per *μ*m^2^ versus the objective lens NA is summarized in the Fig. 1(f), indicating its rather weak variation for the low or moderate focusing conditions (NA = 0.13–0.65). Noteworthy, under tight focusing (NA = 0.95) the pronounced smoothing of the crater topography is observed (see insets in Fig. 1(f)), with the self-organized spiky features arranged mainly around the outer rim of the crater.

Utilization of the moderate-NA focusing conditions is beneficial from the point of view of rapid laser printing. specifically, such conditions provide the longer focal depth ≈*λ*(NA)^−2^ mitigating the sample alignment requirements. Additionally, such loose focusing conditions allow utilization of the ultrafast laser beam scanning with a galvanometric scanning system to take advantage of the MHz repetition rate of the fs laser system used in our experiments (see Methods for details). The example of the large-scale array fabricated for only 2 seconds at a printing rate of 10^5^ craters per second is presented in Fig. 1(g). The distance between the adjacent craters deviates owing to the inaccuracy of the beam positioning system. Meanwhile, such an approach allows ultrafast fabrication of textured surface, when the exact array periodicity is not of mandatory importance.

Previously, we suggested the way to tune the nanotopography inside the spallative crater via its multi-pulse ablation^16^. Meanwhile, in terms of achieving the commercially competitive fabrication rates, implementation of such an approach requires substantially longer fabrication cycle per individual element as well as the ordered array containing millions of such elements. In this way, the single-pulse printing with the unusual laser beams providing the specific intensity (temperature) distribution on the metal film surface can be considered as a more efficient way toward controllable tuning of the surface nanotopography. In this study, we utilized a donut-shape beam^34^ (see Methods, for details) to produce the spallative patterns on the surface of the similar 500-nm thick Ag film (Fig. 1(h)). Series of the SEM images (Fig. 1(h–k)) illustrates the surface textures obtained under irradiation with the focused radially polarized donut-shape beam at increased applied pulse energy. At low pulse energy, the central unmodified area irradiated by the hollow part of the donut-beam can be resolved on the film surface. Noteworthy, the subsequent increase of the pulse energy leads to the formation of the micron-sized tip in the center of the initially blank area via the radially directed movement of the molten material above the unmodified film surface. This case completely differs from those when the central tip appears atop the surrounding nanobump under irradiation with the Gaussian-like laser spot. The later process proceeds via detachment of the entirely molten metal film from the underlying substrate under the action of the thermoelastic stresses. Also, this process requires approximately twice larger pulse energy comparing to the typical value of the spallation threshold measured for such film^16^. The spallative crater with the central tip formed upon irradiation with the donut-shaped beam can be produced at the pulse energy slightly larger than the ablation threshold. Noteworthy, the formation process reproduces from pulse to pulse allowing to print arrays of such structures as illustrated in Fig. 1(l).

We performed comparative analysis of the surface features produced with the Gaussian and donut-shaped beams. This analysis indicates that the structures printed with donut-shaped beam can be characterized by denser nanofeatures per single crater, as the surface near the central micron-high protrusion is not smoothed down by the nanobump. In this way, the direct ablation of the “thermally thick” noble-metal films with donut-shaped beams allows to produce the surface features with multi-scale nanotopography. Namely, the sub-100 nm spikes randomly arranged within the spallative crater as well as the larger spikes on the crater rim can be combined with well-ordered micron-high central protrusions. Such multi-scale topography provides the way to expand the plasmonic response of the textured surface into the infrared spectral region. This potentially allows to simultaneously utilize within the single substrate different sensing approaches as multi-wavelength SERS and SEIRA. Preliminary measurements of the reflection coefficient of the laser textured surfaces using a FTIR spectroscopy (see Fig. S2 in the Supporting information) clearly show some resonant features in the near-IR region. These features can be attributed to the excitation of some resonances of the larger protrusions of the spallative craters as well as the collective effects which come from the periodical arrangement of the craters. The detailed study of the IR plasmonic properties of the laser-textured surface will be discussed in our forthcoming paper.

### RGB color analysis of the scattering from bimetallic textures: tunable visible-range plasmonic response

Recently, we proposed the polarization-resolved RGB color analysis of the plasmon-mediated scattering as an efficient and easy-to-implement method allowing to probe plasmonic response of the chaotically textured surfaces^16^. Here, similar approach was used to provide a comparative spectral-free analysis of the visible-range plasmonic response of the bimetallic textures having variable thickness of the capping Au layer. Spallative craters are covered with the randomly arranged nanospikes, which have high height-to-width (aspect) ratio substantiating their polarization-dependent plasmonic response^16^. Here, for RGB-color analysis we used the side illumination with the s-polarized radiation (see Methods) to predominantly excite the radial oscillations of the electron plasma in the direction which is perpendicular to the major axis of the nanotips. Considering the size and the aspect ratio of the nanospikes, such excitation is expected to be more efficient in the visible spectral range (namely, in its blue region), in agreement with our previous experimental observations^16^. At the same time, similar radial electron plasma oscillation will be excited upon normal irradiation of the surface textures, which will be implemented further to demonstrate biosensing properties of the produced surfaces.

**Figure 2:**
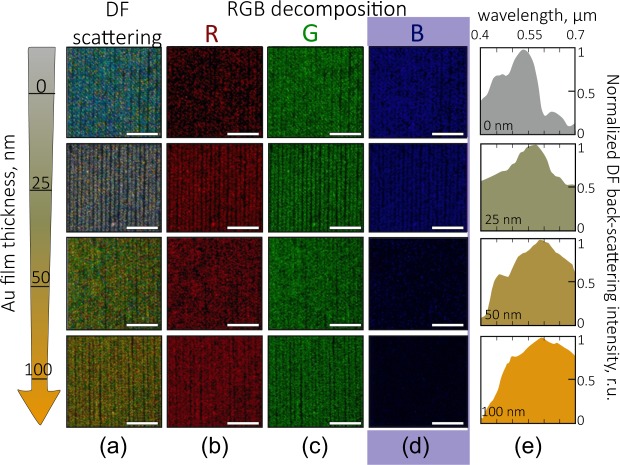
Tuning the plasmonic response of the Ag spallative textures via their decoration with the Au capping layer: RGB color analysis. (**a**) DF optical images of the Ag laser-textured surfaces covered with the Au capping layer of variable thickness ranging from 0 to 100 nm. The images are taken under s-polarized white-light side illumination. For better representation, the intensity of the images is homogenized by varying the image accumulation time. (**b**–**d**) Decomposition of the DF images into the red, green, and blue channels. Scale bar in all images corresponds to the 20 *μ*m. (**e**) Normalized DF back-scattered spectra measured from the corresponding laser-textured areas. Each spectrum was averaged over 10 similar measurements from randomly chosen areas having the size of 50 × 50 *μ*m^2^.

The change of the scattering color on the recorded DF optical images observed for the arrays of the spallative textures upon increasing of the thickness of the capping Au layer clearly indicates the tuning of the corresponding plasmonic response (Fig. 2(a)). By decomposing the obtained DF images into the red, green and blue channels with a computer software (see Methods), one can find the remarkable decrease of the intensity of the blue channel upon increasing of the corresponding Au film thickness (Fig. 2(b–d)). In particular, this indicates that for Au films thicker than 50 nm, the Ag textured surface becomes completely covered by the Au capping layer. This governs a strong drop of the plasmon-mediated scattering in the blue spectral region (Fig. 2(d)). Meanwhile, for relatively thin Au capping layer one can expect the spectrally broadband (over the whole visible spectral range) electromagnetic response, which can be achieved via the decoration of the Ag spallative features with the Au nano-flakes. Such decoration produces the cascaded nanotexturing as well as provides the constructive intermixing of the plasmonic responses from different materials (see Fig. 1(c–e), for example). By directly measuring the scattering spectra from the bare and Au-decorated Ag spallative craters (Fig. 2(e)), we found good qualitative agreement with the estimations based on the RGB decomposition. Particularly, this shows the applicability of the proposed easy-to-implement spectroscopy-free RGB color analysis for prediction of the visible-range plasmonic properties of the textured surfaces.

The intensity of the plasmon-mediated far-field scattering can be considered as an indicator of the enhanced electromagnetic near-fields^35^. In this way, for the fixed excitation conditions we measured the scattering intensity from the bare and Au-decorated laser-textured Ag surfaces to compare qualitatively their intensity and density of the electromagnetic hot spots. The strongest scattering over the whole visible spectral range is observed for the Ag spallative craters capped with the 25-nm thick Au layer (see Fig. S3 in the Supporting information), apparently owing to the formation of the cascaded nanotextured surface producing multiple hot spots. These measurements were performed with two objective lenses (NA = 0.42 and 0.8), allowing to vary the collection angle for the scattered light. For both these lenses, we observe a similar trend indicating that the enhanced signal for the Ag-Au bimetallic textures is not related to the directional scattering.

To end up this section, along with the pronounced cascaded topography the deposition of the Au film will expectedly red-shift the plasmonic response of the whole substrate. Meanwhile, to produce the strong field enhancement in the blue spectral region, the other materials as Al, Pt and Pd exhibiting their plasmonic response in this spectral region can be coated onto the printed textures using similar physical vapor deposition procedure. To illustrate this, we produced Ag-Pt and Ag-Al bimetallic surface textures fabricated using similar procedure (see Fig. S1 in the Supporting information).

### FDTD calculations of the near-field EM enhancement

**Figure 3:**
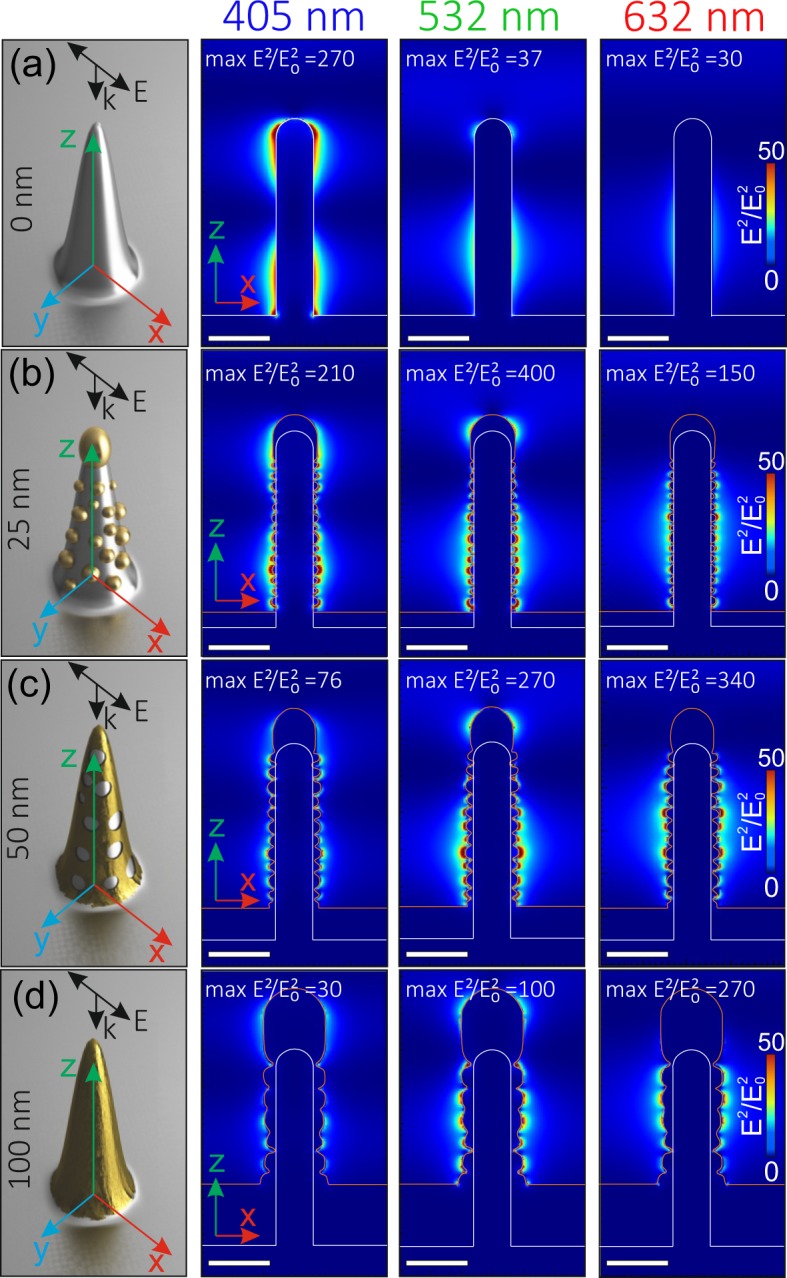
Squared normalized electric field amplitude (E)^2^/(E_0_)^2^ calculated near bare (**a**) and Au-coated (**b**,**c**) isolated Ag nanospikes at various excitation wavelengths of 405, 532 and 632 nm. The left-most column shows the schematic representation on the simulated geometry. Orange and blue curves in the calculated distributions indicate the gold and silver contours. The maximal value of the color bar is fixed at 50 for all maps for better representation, while the maximal (E)^2^/(E_0_)^2^ value reached at the specific points is indicated in each distribution. Scale bar corresponds to 100 nm.

Supporting FDTD calculations were performed to evaluate the enhancement of the electromagnetic fields near the bare and Au-decorated silver spikes for the three fixed excitation wavelengths, covering the entire visible spectral range (see Methods for details). The calculation results for the excitation wavelengths of 405, 532 and 632 nm and the excitation of the nanospike from the top are summarized in Fig. 3. According to our calculation, bare Ag spike provides considerable enhancement only at 405 nm, while substantially smaller values of the squared EM-field amplitude can be reached in the green and red spectral ranges Fig. 3(a). The decoration of the Ag tip with the Au nano-flakes upon deposition of the 25-nm thick Au layer provides highly textured surface allowing to get strong EM enhancement at all three excitation wavelength (Fig. 3(b)). Further increase of the thickness of the Au capping layer results in a gradual reduction of the EM-field enhancement at 405 nm (Fig. 3(c,d)), which is in a good agreement with the performed RGB color analysis of the plasmon-mediated scattering (Fig. 2).

As the nanospikes can be characterized by the broad deviation of the size parameters even within a single spallative crater, the performed FDTD analysis is rather approximate in terms of assessment of the maximal EM enhancement. By performing similar calculations for different sizes of the Ag tip, one can get different geometry-dependent values of the maximal enhancement of the squared EM-field amplitude, besides the previous general trend demonstrating the broadband performance of the bimetallic Ag-Au nanospikes remains the same. Here, owing to limitation of the computation resources for fine mesh simulations, the EM calculations were performed for the isolated nanospike. Meanwhile, their dense arrangement within the spallative crater (≈15–20 spikes per *μ*m^2^) ensures the plasmonic inter-coupling between neighboring structures. Such coupling is expected to work even under excitation with a tightly focused laser radiation (say, with 0.9-NA lens) as well as is expected to increase the overall EM enhancement inside the crater.

### Multi-wavelength SES performance of the bimetallic laser-printed textures

The observed strong and spectrally broadband plasmonic response of the laser-printed Ag spallative textures decorated with the Au nano-flakes provides favorable conditions for their implementation for multi-fold enhancement of SERS and SEPL signal from various quantum emitters. In terms of SEPL applications, the textured substrates supporting broadband plasmonic response are beneficial as the maximal PL yield was reported for the substrates with the plasmonic response fitting both the emission and absorption bands of the quantum emitter placed in the vicinity of the plasmon-mediated hot spot. To illustrate the applicability of our substrates for multi-wavelength SEPL enhancement, we first probed the PL yield from the nanometer-thick R6G layer pumped at variable excitation wavelengths (see Methods for details).

**Figure 4:**
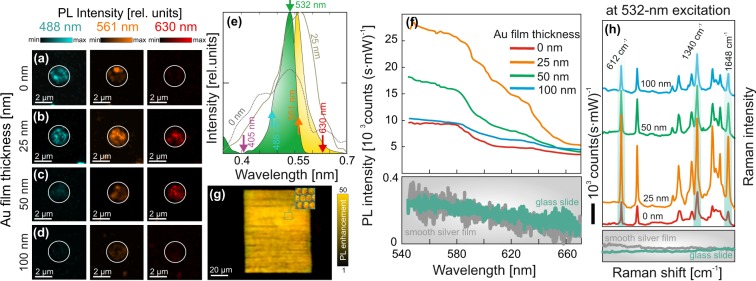
(**a**–**d**) Confocal R6G PL images of the single laser-textured craters with variable thickness of the Au capping layer ranging from 0 to 100 nm obtained under various excitation wavelength of 488, 561 and 630 nm. Images are presented in false colors. The white circles indicate the position of the isolated crater. The excitation laser intensity for the fixed wavelength is a constant value. (**e**) Averaged DF back-scattering spectra of the Ag and Ag-Au laser-printed textures (dashed curves) superimposed with the R6G absorption (green) and emission bands (yellow). Intensity level of the back-scattering spectra indicates the relative difference between the scattering intensity measured for mono- and bimetallic surfaces. The arrows indicate the excitation wavelengths used in the SEPL experiments. (**f**) R6G SEPL spectra measured on the laser-textured surfaces at variable thickness of the Au capping layer. Each presented spectrum was averaged over 50 similar spectra taken from the randomly chosen textured structures. The averaged SEPL signals obtained from the smooth silver film and glass substrates are given for comparison on the bottom panels. (**g**) R6G SEPL image of the bimetallic textured surface fabricated using ultrafast scanning with the galvanoscanner. Magnified section showing the arrangement of the craters in the textured area is given in the inset. (**h**) R6G SERS spectra measured on the laser-textured surfaces with a variable thickness of the Au capping layer. Several R6G Raman bands used to estimated the Raman yield are highlighted. Each presented spectrum was averaged over 50 similar spectra taken from the randomly chosen textured structures. The averaged R6G SERS signal obtained from the smooth silver film and glass substrates are given for comparison on the bottom panels.

The confocal PL images of the R6G nanometer-thick layer covering isolated mono- and bimetallic craters are presented in Fig. 4(a–d) revealing the general tendency predicted by the RGB color analysis and FDTD calculation. In particular, the isolated pure Ag surface spiky texture demonstrates the strong PL enhancement under excitation at the 488-nm laser radiation with much weaker PL yield at 561 nm excitation dropping to almost zero-level at 630 nm. At the same time, under the same excitation conditions, the Ag texture capped with 25-nm thick Au layer shows substantial R6G PL enhancement at all probed excitation wavelength substantiating the broadband plasmonic response of the bimetallic textures. Noteworthy, a gradual increase of the thickness of the Au capping layer results in a considerable drop of the PL yield, at first, under 488-nm excitation and then - at all the pumping wavelength. The R6G PL excitation at 405-nm wavelength was also implemented showing the noise level signal for both the mono- and bimetallic substrates, particularly owing to the extremely low R6G excitation efficiency at such conditions (see Fig. 4(e)).

To further study the R6G SEPL enhancement on the laser-textured substrates, we used the 532-nm excitation wavelength, which coincides with the R6G PL excitation maximum, and detected the PL spectrum from the single texture with a home-build microspectroscopy setup. The pronounced enhancement of the spontaneous emission from the R6G molecules coated above the laser-textured surfaces was obtained with maximal averaged SEPL yield of 27 ± 5 ⋅ 10^3^ counts (s⋅mW)^−1^ observed for bimetallic Ag-Au textures with the 25-nm thick Au capping layer (Fig. 4(f)). Noteworthy, such “optimal” bimetallic textured surface provide ≈3 times more intense SEPL yield being compared to the pure Ag spallative textures and about 75-fold signal enhancement being compared to the smooth Ag film (or glass surface) under similar irradiation and signal collection conditions (see bottom chart in Fig. 4(f)). Noteworthy, the difference between the SEPL enhancement for the mono- and bimetallic textures is expected to be more pronounced for the excitation wavelength in the yellow-red part of the spectrum, as one can see from the multiwavelength confocal images (Fig. 4(a–d)). Generally, the observed R6G SEPL enhancement for the Ag-Au self-organized textures can be attributed to the contribution of several factors. First, one could mention an increased density of the plasmon-mediated electromagnetic hot spots as well as the maximal EM enhancement achieved for the cascaded nanotopography (which was found to be optimal for 25-nm thick Au capping layer). Second, such textures provide twice faster R6G emission rate via reduced lifetime^16,36^. Finally, the Au-decorated textures provide matching of their plasmon-mediated spectrum with the absorption/emission bands of the R6G molecules, particularly, in the red-orange part of the spectrum. Noteworthy, the latter facilitates more efficient interaction between the plasmonic nanotextures and attached emitters^37–40^. This feature is confirmed by the corresponding deformation of the R6G emission spectrum manifested in the increasing intensity in its orange part for bimetallic textures being compared to the R6G emission enhanced by the bare Ag textures (see Fig. S4 in Supporting information). As it was mentioned, the biosensing textured surfaces representing the arrangements of the spiky craters can be produced on the surface of the Ag film using the ultrafast galvanometric scanning of the laser beam. Figure 4(g) shows the example of the bimetallic nanotextured area with the size of 80 × 100 *μ*m^2^ printed in such manner. The textured surface demonstrates the pronounced and rather uniform SEPL enhancement of the covering R6G layer being compared to the non-textured surface. The enhanced electromagnetic fields of the bimetallic textures were also probed by detecting the R6G SERS spectra (see Methods) as the Raman yield scales with the EM-field amplitude in the produced “hot spots”. By accounting for the average intensity of the several R6G Raman bands at 612, 1360 and 1648 cm^−1^ we found more than an order of magnitude higher SERS yield for Ag-Au bimetallic textures being compared to the monometallic ones (see Fig. 4(h)). The averaged SERS enhancement factor for such bimetallic texture was estimated to be ≈1.5 ⋅ 10^6^ at 532-nm wavelength.

## Conclusions and outlook

Here, an easy-to-implement ultrafast direct laser printing via spallation of thermally thick silver films and subsequent large-scale magnetron deposition of nanometer-thick Au layer was implemented to produce bimetallic textured surfaces with the cascaded nanotopography. The produced bimetallic textures demonstrate the strong broadband plasmonic response over the whole visible spectral range, which was confirmed by our supporting calculations of the EM near-fields, as well as by comprehensive optical characterization via the DF scattering microspectroscopy and the RGB color analysis of the DF scattering images. The produced bimetallic laser-printed nanotextures were shown to provide enhancement of the SEPL (up to 75-fold) and SERS (up to 10^6^) yields for the organic dye molecules pumped at various excitation wavelengths, making the designed and demonstrated spectrally-broadband textures promising for routine biosensing applications. Additionally, comprehensive optical and biosensing characterization of the laser-printed bimetallic surface structures allows to substantiate the convinient spectroscopy-free RGB color analysis as a valuable tool for predictive assessment of the plasmonic properties of various chaotically and quasi-regularly nanotextured surfaces.

Laser spallation, as an initial step to produce such nanotopographies, can be applied for pure plasmonic materials (as Ag, Au, Pd, Pt and Al) as well as their multicomponent alloys. When combined with the subsequent deposition of the nanometer-thick plasmonic capping layer, ultrafast laser printing provides the novel platform to design nanotextured surfaces with complex surface distribution of the corresponding chemical elements. It is worth noting that utilization of the multi-metallic alloyed textures can be beneficial in terms of advanced nanotopography and efficient intermixing of the plasmonic responses from different materials^41^. Moreover, such alloyed textures can also provide superior biosensing performance via chemical enhancement mechanisms^42^, which is to be studied in our forthcoming paper.

## Electronic supplementary material

Supplementary information
