# Supplementary material for: Ultrafast laser printing of self-organized bimetallic nanotextures for multi-wavelength biosensing
Source: Sci Rep. 2018 Nov 7;8:16489. doi: 10.1038/s41598-018-34784-y (PMC6220284; doi:10.1038/s41598-018-34784-y)
Supplement: Supplementary file 1 — Supplementary information [file 41598_2018_34784_MOESM1_ESM.pdf]

Supporting Information

Ultrafast laser printing of bimetallic self-organized nanotextures for multiwavelength biosensing

D. Pavlov[1,2], S. Syubaev[1,2], A. Cherepakhin[2], A. Sergeev[1,2], O. Vitrik[1,2], A. Zakharenko[1], P. Danilov[2,3], I. Saraeva[3], S. Kudryashov[2,3,4], A. Porfirev[2,5,6], A. Kuchmizhak[1,2,*]

1 School of Natural Sciences, Far Eastern Federal University, Vladivostok, Russia

2 Institute of Automation and Control Processes, Far Eastern Branch, Russian Academy of Science, Vladivostok 690041, Russia

3 Lebedev Physical Institute, Russian Academy of Sciences, Moscow 119991, Russia

4 ITMO University, St. Peterburg 197101, Russia

5 Samara National Research University, 34 Moskovskoe Shosse, Samara 443086 Russia

6 Image Processing Systems Institute of the RAS-Branch of FSRC ``Crystallography \& Photonics'' of the RAS, 151 Molodogvardeyskaya St., Samara 443001, Russia

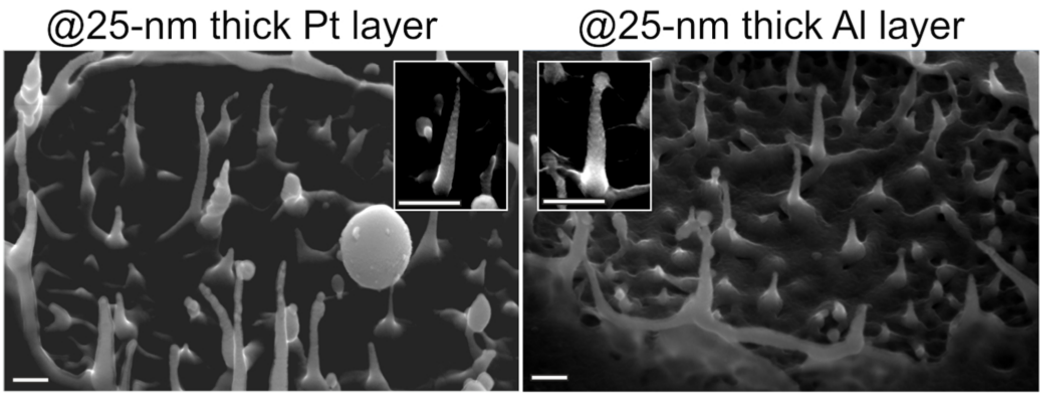

Figure S1. Side-view SEM images of the Ag textured surfaces capped with a 25-nm thick Pt (left) and 25-nm Al layers (right). Insets show magnified view of the isolated bimetallic spiky feature. Scale bars correspond to 200 nm.

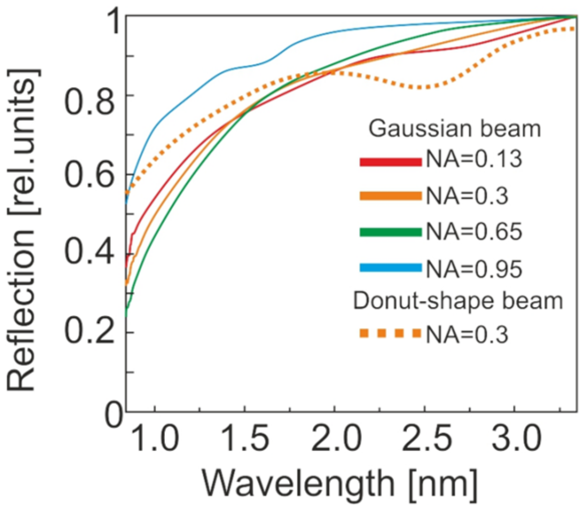

Figure S2. Normalized FTIR spectra measured from the Ag textured surfaces printed at various focusing conditions (defined by the lens NA) using Gaussian- (solid curves) and donut-shaped beams (dotted curve).

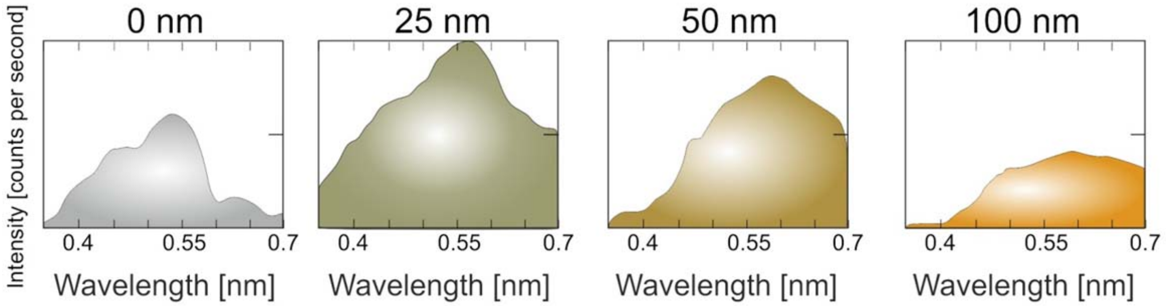

Figure S3. Averaged DF back-scattering spectra measured from the laser-textured Ag textures capped with Au films of variable thickness. Each spectrum was averaged over 10 similar measurements from randomly chosen areas having the size of 50x50 μm^2. The strongest scattering is observed for Ag-Au textures at 25-nm thick Au capping layer indicating the maximal density and enhancement of the electromagnetic hot spots.

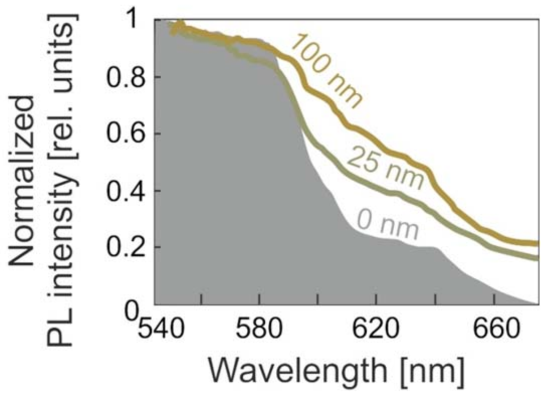

Figure S4. Normalized R6G SEPL spectra on the laser textured Ag surface covered with the Au layer of variable thickness showing deformation of the R6G emission spectra for bimetallic textures.
